# Canis familiaris allergen Can f 6: expression, purification and analysis of B-cell epitopes in Chinese dog allergic children

**DOI:** 10.18632/oncotarget.21822

**Published:** 2017-10-13

**Authors:** Yu-Jie Wang, Lin Li, Wei-Juan Song, Yan-Jun Zhou, Meng-Da Cao, Xiang-Rong Zuo, Ji-Fu Wei

**Affiliations:** ^1^ Research Division of Clinical Pharmacology, The First Affiliated Hospital of Nanjing Medical University, Nanjing, Jiangsu, China; ^2^ Department of Critical Care Medicine, The First Affiliated Hospital of Nanjing Medical University, Nanjing, Jiangsu, China; ^3^ Department of Clinical Laboratory, The First Affiliated Hospital of Nanjing Medical University, Nanjing, Jiangsu, China; ^4^ Department of Emergency Medicine, The First Affiliated Hospital of Nanjing Medical University, Nanjing, Jiangsu, China

**Keywords:** Can f 6, dog allergy, epitopes, allergenicity, Chinese children, Immunology and Microbiology Section, Immune response, Immunity

## Abstract

Dog allergy is common worldwide. However, the allergenicity of dog allergy is still unclear in China as well as in special group, such as children. In this study, we chose Can f 6, a major dog allergen which belongs to the lipocalin to study its allergenicity in Chinese dog allergic children. Can f 6 gene was subcloned into pET-28a vector and transformed into *E. coli* BL21 (DE3) cells for expression. The recombinant Can f 6 was purified by nickel affinity chromatography, identified by SDS-PAGE, and tested for its allergenicity by Western blot with sera and basophil activation test. Secondary structures, B cell epitopes and homology modeling of Can f 6 were predicted by using a series of bioinformatical approaches. And the verification of B cell epitopes was detected by ELISA. The recombinant allergen showed an explicit band with the molecular weight of 20 kDa by SDS-PAGE. Sera from 56.3 % (18/32) of dog-allergic children patients reacted with Can f 6. The induction of the expression of CD63 and CCR3 of dog allergic children in passively sensitized basophils was up to approximately 5.0 times higher than healthy subjects. The secondary structure of Can f 6 contains 3 α-helices, 9 β-sheets and random coils. Five B cell epitopes of Can f 6 were predicted and were confirmed successfully by ELISA. The results showed Can f 6 is a major allergen in Chinese children, which provides a basis for further study of Can f 6 in diagnosis and treatment of symptoms in children in China. The structural information of Can f 6 will help to form a foundation for the future design of vaccines and therapies for Can f 6 related allergies.

## INTRODUCTION

Dogs are the predominant allergic source worldwide, which can provoke severe allergic disease, such as asthma, urticaria and rhinitis [1]. Domesticated dogs initially served as a practical character for protection or agricultural use in human being, but served a more emotional role of companionship recently. With this paradigm shift, dogs begin to move indoors. And this unremitting exposure is associated with the progress and deterioration of dog allergic disease. For the patients with dog allergic disease, avoidance is clearly the best treatment modality. But this does frequently not occur, even in settings beyond occupational allergy. The sensitization rate to dogs was reported as high as 10 % of all people in Western countries, varying by country, area, time period, and atopic predisposition [2, 3].

Dog dander is the common source of dog allergens, which includes members of dog albumin Can f 3 and lipocalin protein family members, Can f 1, Can f 2, Can f 4 [4, 5, 6, 7] and Can f 6 [8]. Can f 1 [9] as well as Can f 2 have also been detected in the dog saliva [2]. Recently, a major dog allergen, which belongs to the kallikrein, Can f 5, was discovered in dog urine, suggesting urine and saliva as very important allergen sources [10].

Since identified in 2011, the dog allergen Can f 6, one lipocalin protein, was rarely studied worldwide. The sensitization (%) of Can f 6 listed in the WHO/IUIS allergen database (www.allergen.org) is 61 % [11]. In 2012, Nilsson *et al* reported that 38 % (38/100) of the dog-sensitized Swedish subjects were positive to Can f 6 [8]. However, there are few reports about the sensitization of Can f 6 in other countries. With the rapid increase of the number of the pet dogs, some typical allergic diseases caused by dog allergens, such as asthma, atopic dermatitis and eczema, are becoming more frequent in China. However, few studies were conducted in dog allergens in China. In addition, considering the frequent contacts with the pet dogs, children’s morbidity of allergic disease is getting higher. A high proportion (47.32 %) of Chinese children with early allergic symptoms developed respiratory allergies in their early school years [12]. Similarly, there are still few reports about children’s allergy to dog allergens in China.

Identification of 3D structure of allergen can be used for understanding the immune response of allergenic proteins, including the cross-reactivity among homologous allergens. B cell epitopes show fundamental differences in the localisation on allergen molecule and in eliciting the immunological responses as well. B cell epitopes can be linear or conformational and they are located on the surface of the allergen usually for binding easily with antibody molecules.

Due to the highest sensitization rate in dog allergens by the lipocalin protein, we expressed Can f 6 and studied its allergenicity in Chinese dog allergic children by the western blot and basophil activation test. We also used bioinformatical approaches to predict the secondary structures, B-cell epitope and homology modeling of Can f 6 in the present study. And the B cell epitopes were identified by ELISA with sera of Chinese Can f 6 allergic children. These results will provide a basis for improved diagnosis and treatment of dog allergy in Chinese children.

## RESULTS

### Optimized codon

**Figure 1:**
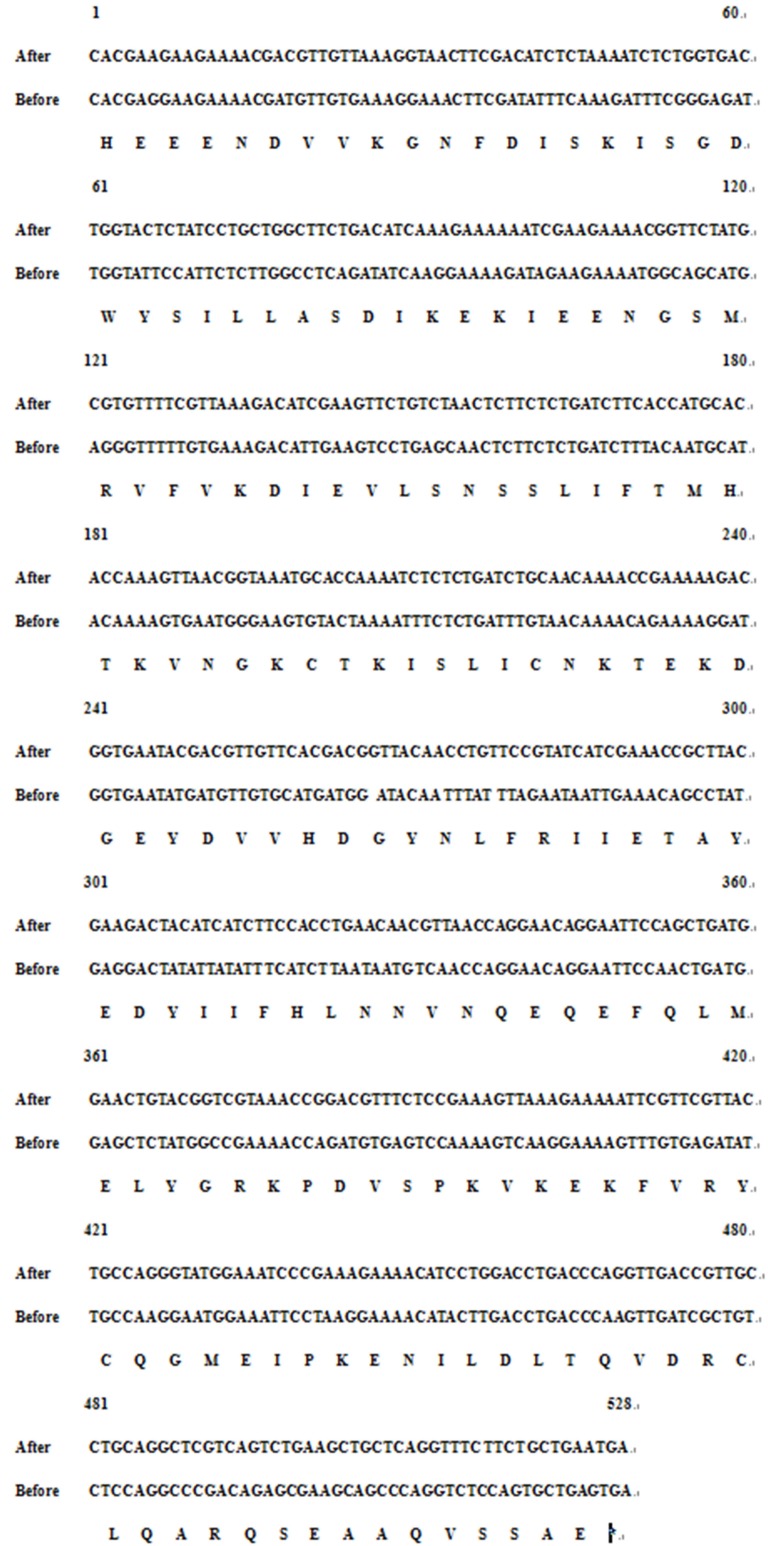
The nucleotide sequences for codon-optimized Can f 6 gene before and after codon optimization

The optimized codon to *E.coli* of Can f 6 is showed in Figure 1.

### Expression and purification of Can f 6 in *E.coli*

**Figure 2:**
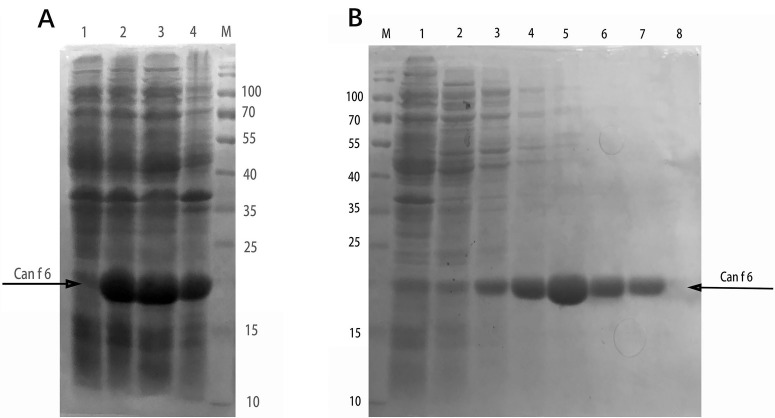
Expression and purification of Can f 6 in *E. coli* **A.** Lane M, protein molecular weight standard; Lane 1, non-induced recombinant Can f 6 whole cell lysate; Lane 2, IPTG-induced recombinant Can f 6 whole cell lysate; Lane 3, supernatant fraction after ultrasonication; Lane 4, precipitation fraction (inclusion bodies) after ultrasonication. The Can f 6 was denoted with an arrow; **B.** SDS-PAGE after affinity chromatography of Can f 6. Lane M, protein molecular weight standard; Lane 1, Unbinding fractions of the supernatant of Can f 6; Lane 2, washing with 10 mM imidazole; Lane 3, washing with 50 mM imidazole; Lane 4, washing with 100 mM imidazole; Lane 5, washing with 150 mM imidazole; Lane 6, washing with 200 mM imidazole; Lane 7, washing with 250 mM imidazole; The arrow point to the purified Can f 6.

The codon optimized of Can f 6 sequence was subcloned into pET-28a (+) vector and transformed into BL21 (DE3) pLysS *E. coli.* A comparison of sequences before and after optimization is shown in Figure 1. By the SDS-PAGE, it was discovered that Can f 6 was expressed mainly in the supernatant (Figure 2A). Then, the recombinant Can f 6 was purified by Ni column. The purified Can f 6 was showed as a single band identified by SDS-PAGE. The apparent molecular weight of purified Can f 6 was 20 kDa (Figure 2B).

### Immuno-reactivity to IgE of Can f 6

**Figure 3:**
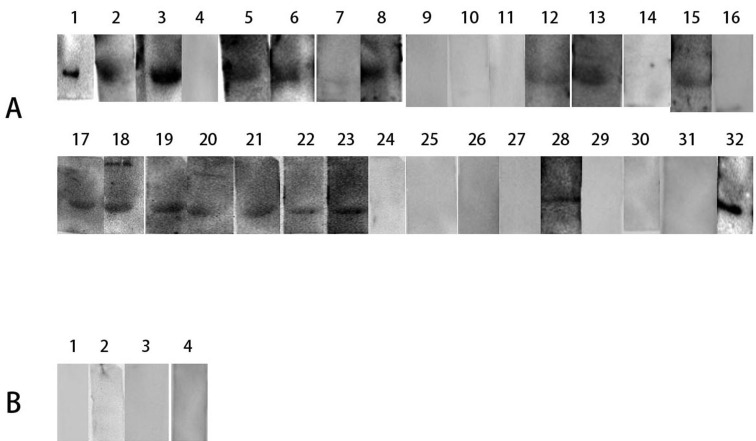
Immuno-reactivity to IgE of Can f 6 Immuno-reactivity to IgE of Can f 6 was identified by western blot analysis. **A.** Can f 6 were incubated with sera of dog allergic patients as the first antibody. **B.** Can f 6 were incubated with sera from 4 healthy individuals as a negative serum control.

**Figure 4:**
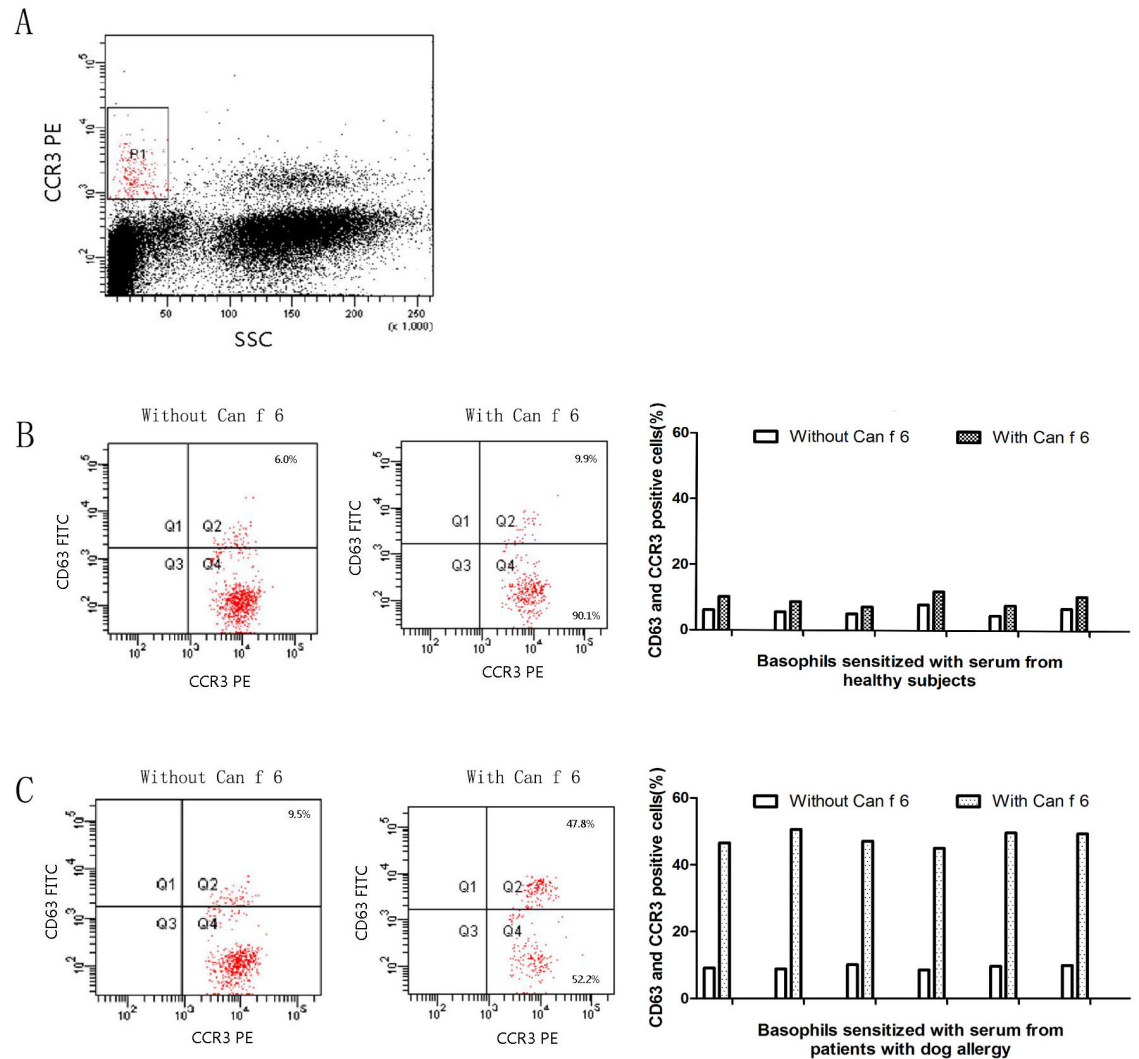
Induction of basophil activation by purified Can f 6 After non-specific IgEs on basophils being removed, cells from each donor were sensitized with sera from 6 healthy subjects or 6 dog allergic patients, and were challenged with Can f 6 at 1.0 µg/ml. The values shown are mean ±SEM for the sera from 6 different subjects. **A.** Gating strategy of the basophil by CCR3 and SSC. **B.** The results of 6 healthy subjects’ sera. **C.** The results of 6 dog allergic patients’ sera.

In order to determine the allergenicity of Can f 6, the ability of Can f 6 to bind specific IgE in the dog-allergy children’s sera were detected by western blot analysis. Totally 32 pediatric dog-allergy patients were included. As Figure 4 shown, 18 of 32 dog-allergy sera of pediatric patients showed positive IgE reactivity to Can f 6, but healthy controls failed to. (Figure 3)

### Basophil activation analysis

In comparison with the healthy control, Can f 6 induced approximately up to 5.0 folds increase in CD63 and CCR3 (Figure 4).

### Homology modeling

**Figure 5:**
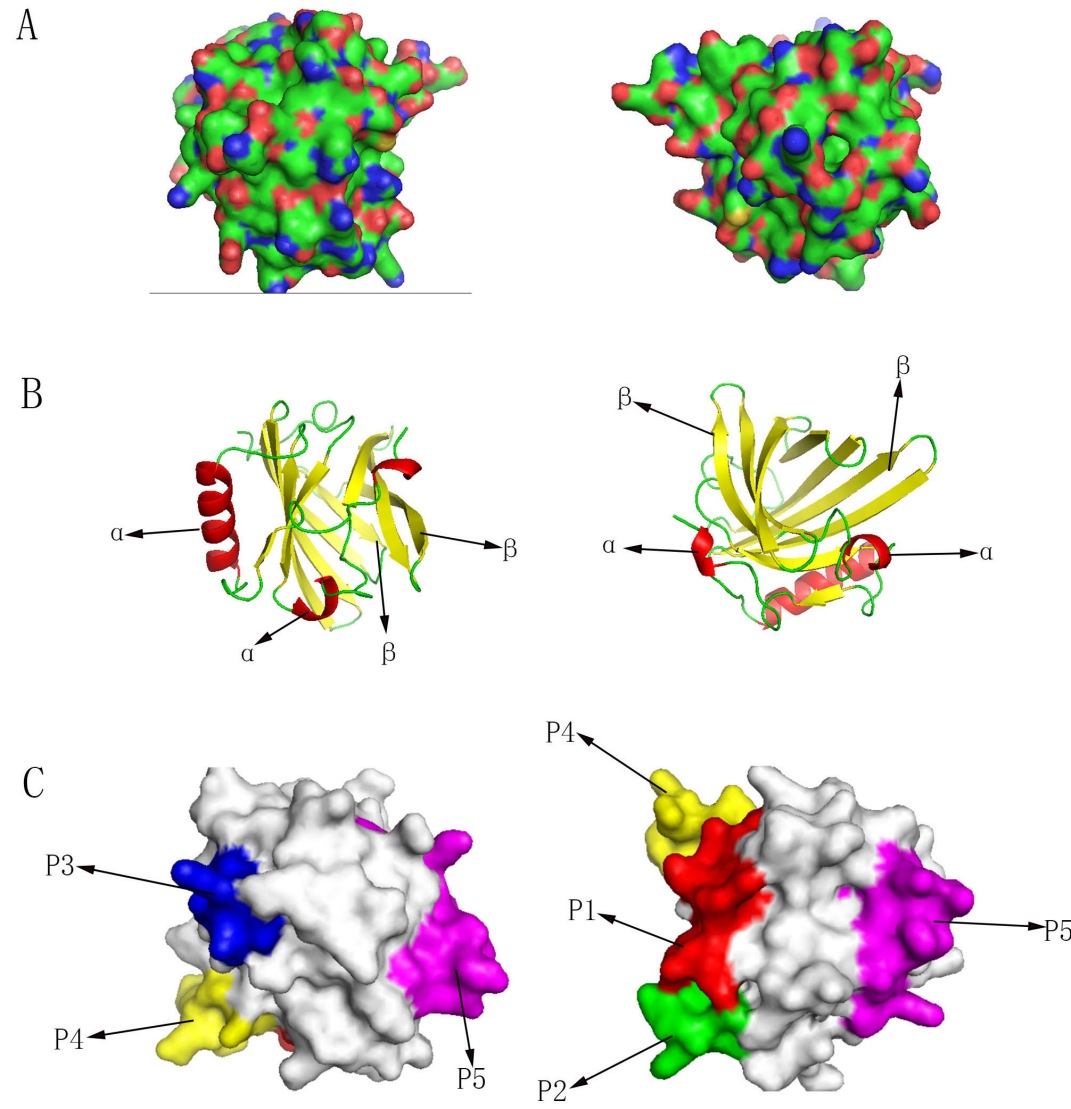
3D structure of Can f 6 **A.** Protein structures of Can f 6 homology model. **B.** Predicted secondary structure showed on the surface of Can f 6 structure. The red symbolized α-helices and the green symbolized β-sheets marked with arrows. **C.** Predicted B-cell epitopes showed on the surface of Can f 6 structure marked with arrows. P1 (Marked with red): SDIKEKIEENGS; P2 (Marked with green): TKVNGKCT; P3 (Marked with blue): KTEKDGE; P4 (Marked with yellow): NVNQEQEF; P5 (Marked with pink): GRKPDVSPKVKEKF.

Looking for the proteins with known 3D structure in the PDB, 1gm6.1.A (PDB accession number) was showed the highest sequence identity (60.69 %) to Can f 6. So, the template 1gm6.1.A was chosen for homology modeling. Figure 5A shows the overall 3D structure of Can f 6. (Figure 5A)

### Secondary structure prediction

The Can f 6 was predicted to contain no transmembrane helices by TMHMM 2.0. Secondary structure prediction showed that Can f 6 contained 3 α-helices, 9 β-sheets and random coils. (Figure 5B) Based on the deduced amino acid sequence with 175 residues, these secondary structure comprises α-helices 21/175 (12 %), β-sheets 77/175 (44 %), and random coils 77/175 (44 %).

### B-cell epitopes prediction

Based on the four properties (hydrophilicity, flexibility, accessibility, and antigenicity), the final predicting regions of Can f 6 allergen by DNAstar were obtained as: 43-54, 91-97 and 139-152. The predicted results of BPAP system were 43-54, 76-83, 91-97 and 125-132. And the predicted results of BepiPred 1.0 server were 16-24, 43-54, 125-132 and 139-152. Furthermore, the final potential B cell epitopes of Can f 6 were selected on the basis of the results of these three tools. The ultimate results of the three immunoinformatics tools finally predicted 5 B-cell epitopes in Can f 6: P1 (Marked with red): SDIKEKIEENGS; P2 (Marked with green): TKVNGKCT; P3 (Marked with blue): KTEKDGE; P4 (Marked with yellow): NVNQEQEF; P5 (Marked with pink): GRKPDVSPKVKEKF. (Table 2, Figure 5C).

**Table 1:** Information of patients.

No.	Gender	Age	ImmunoCAP(KU/L)		Associated Disease
1	Female	1	0.96	Asthma	
2	Female	1	59.6	Asthma	
3	Male	2	2.16	Pollinosis	
4	Female	3	1.17	Asthma	
5	Female	3	91.7	Asthma, Rhinitis	
6	Female	4	5.61	Rhinitis	
7	Female	5	13.8	Asthma, Rhinitis	
8	Female	6	2.61	Cough	
9	Female	6	21.1	Rhinitis	
10	Male	6	56.1	Asthma, Rhinitis	
11	Female	6	15.3	Asthma, Rhinitis	
12	Female	7	3.84	Asthma, Rhinitis	
13	Female	7	7.87	Rhinitis,Gasp,Eczema	
14	Male	7	31.3	Rhinitis	
15	Male	7	18.4	Asthma	
16	Female	7	9.85	Asthma, Rhinitis	
17	Female	8	24.5	Cough	
18	Female	8	>100	Asthma	
19	Female	8	1.79	Chest Distress	
20	Male	8	1.43	Rhinitis, Cough	
21	Female	8	3.17	Rhinitis	
22	Male	9	17.3	Rhinitis	
23	Male	9	0.73	Rhinitis	
24	Male	9	63.6	Asthma	
25	Female	10	77.4	Asthma	
26	Male	10	1.54	Gasp	
27	Female	10	20.2	Dermatitis	
28	Female	11	1.52	Rhinitis	
29	Female	11	13.9	Asthma, Rhinitis	
30	Male	11	0.92	Asthma, Rhinitis	
31	Female	12	8.57	Asthma, Rhinitis	
32	Female	12	36.6	Rhinitis	

**Table 2:** Location of the B cell epitopes of the Can f 6 in *Canis familaris* predicted by immunoinformatics tools.

Type of epitope	Peptide	Position	Sequence
B	P1	43-54	SDIKEKIEENGS
	P2	76-83	TKVNGKCT
	P3	91-97	KTEKDGE
	P4	125-132	NVNQEQEF
	P5	139-152	GRKPDVSPKVKEKF

### B-cell epitopes verification

**Figure 6:**
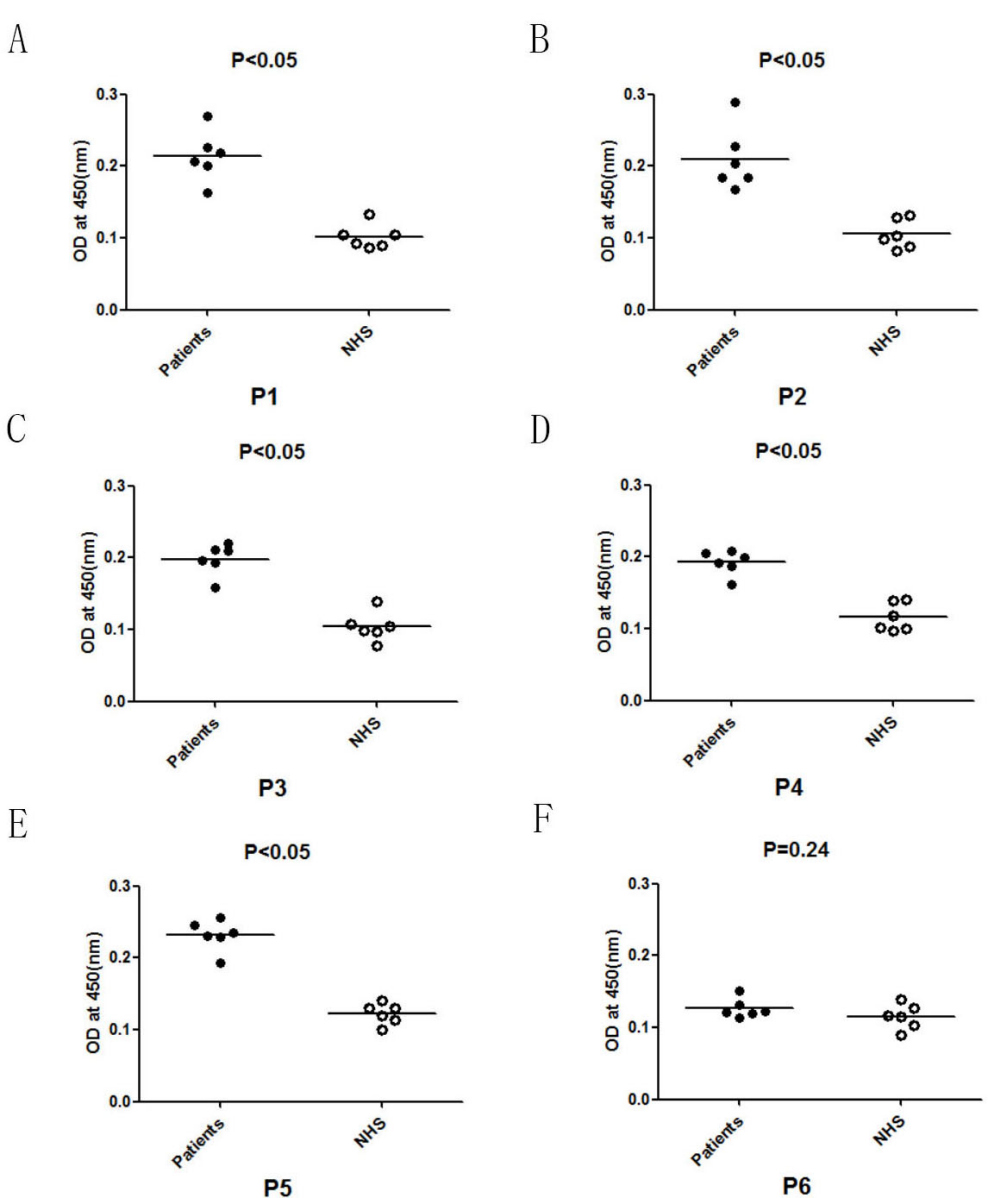
IgE binding studies of B cell peptides of Can f 6 with the dog allergic children’s sera and NHS by ELISA **A.** The results of dog allergic children’s sera compared to the normal human serum (NHS) of the first B cell epitope (P1). **B.** The results of dog allergic children’s sera compared to NHS of P2. **C.** The results of dog allergic children’s sera compared to NHS of P3. **D.** The results of dog allergic children’s sera compared to NHS of P4. **E.** The results of dog allergic children’s sera compared to NHS of P5. **F.** The results of dog allergic children’s sera compared to NHS of an unrelevant control peptide (P6) from the Can f 6 sequence.

The specific IgE binding of predicted B cell peptides was detected by ELISA using dog allergic children’s sera (*n* = 6). The results of all peptides (P1, P2, P3, P4, P5) of patients was about OD = 2 times of the control NHS (Normal Human Serum). Among 5 B cell peptides, all of the peptides reacted with the 6 dog allergic children’s sera and showed significantly different IgE binding (*p* < 0.05) compared to NHS. However, to the unrelevant control peptide (P6), there is no significantly different IgE binding (*p* = 0.24) compared to NHS. (Figure 6)

### Inhibition ELISA

**Figure 7:**
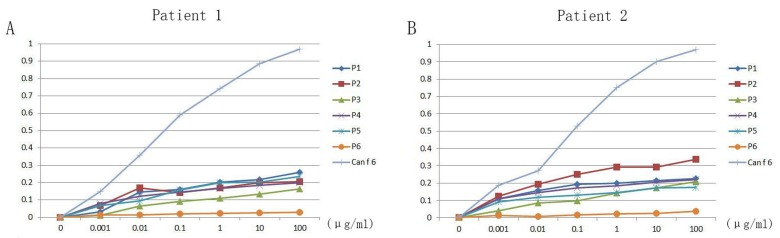
Inhibition ELISA experiments of Can f 6 with 5 B-cell peptides, an unrelevant control peptide and recombinant Can f 6 in two dog allergic children **A.** The results of inhibition experiment in patient 1. **B.** The results of inhibition experiment in patient 2.

Inhibition ELISA experiments were detected with 5 B cell peptides, an unrelevant control peptide (P6) and recombinant Can f 6 in two dog allergic children. All of five predicted B cell peptides could inhibit the binding of two patients’ IgE antibodies to the precoated Can f 6. Maximal inhibition by P1-P5 was about 20% in patient 1 (Figure 7A), whereas 100% inhibition could be achieved by recombinant Can f 6, and there is no inhibition by P6. In patient 2 (Figure 7B), maximal inhibition by P1 was about 30%, and about 20% by P2-P5. 100% inhibition could be achieved by recombinant Can f 6, and there is no inhibition by P6 as well.

## DISCUSSION

To better understand the Can f 6-mediated dog allergy in Chinese children, we prepared highly pure Can f 6 in this study, which is the key step to diagnose and treat the dog allergic disease [13, 14]. Can f 6 was expressed mainly in the supernatant in *E. coli* and purified successfully by Ni column. Can f 6 has immunological activities to bind IgE in the dog allergic children’s sera and 18 in total 32 sera were positive. The sensitization rate we obtained from this study was 56.3 %, proving that Can f 6 was a major allergen of the dog in Chinese children. This is just an initial evaluation of its IgE-binding ability as an allergen in dog-allergic children with allergic symptoms in China. In addition, basophil activation test we carried out herein was a more advanced technique for the test of the allerginicity to a given allergen. In the test, we confirmed that Can f 6 was an active allergen because it could activate basophils which were sensitized by the dog allergic sera. Compared to the previous studies on Can f 6, the innovation of our experiment is that we analyzed the Can f 6 allergies on Chinese children, and the sensitization rate we obtained was close to the foregoing report [11] (56.3 % to 61 %), which indicated there is no significant difference between adults and Chinese children. The result suggests that Can f 6 is also a major allergen among Chinese children. Considering the increase of the number of pet dogs in China, it is very crucial to analyze the situation of dog allergens and this experiment provides a reference for the study of other allergens.

The α-helices and β-sheets are two major secondary structures of protein, whose structures are maintained by hydrogen bonds, making it improbable for epitope sequences to be located in them. Inversely, random coils always contain epitope sequences because they are located in the surface-exposed region [15]. The secondary structures of Can f 6 were predicted to contain 3 α-helices, 9 β-sheets. Based on the deduced amino acid sequence with 175 residues, the secondary structure comprises α-helices 21/175 (12 %), β-sheets 77/175 (44 %), and random coils 77/175 (44 %). The 44 % of residues consist of random coil, which may be linked to some particular characters of Can f 6. The analysis of the structure and function of Can f 6 will contribute to the potential utility in a peptide-based vaccine design for dog allergy.

In addition, we analyzed the B-cell epitopes of Can f 6 and drew them out on the 3D structure of Can f 6. The synthesis of allergen-specific IgE is the key step for the progress of allergic symptoms. The generation of IgE needs B cells to undergo switch recombination in close contact with allergen-specific T helper 2 cells (Th2) [16]. So, the seeking of epitopes was very crucial to allergic symptoms. *In silico* prediction has already become a useful method for identifying epitopes from immunologically associated proteins [17]. Hydrophilicity, antigenicity, segmental mobility, flexibility and accessibility have been used to predict B-cell epitopes on proteins’ sequence, which based on the propensity values of amino acid natures in many algorithms [15]. In this study, we predicted 5 peptides (P1-P5) as potential B-cell epitopes of Can f 6. In addition, we tried to confirm the allergenicity of the predicted B cell epitopes by ELISA. And the results showed significant differences statistically between Chinese dog allergic Children and the NHS (*P* < 0.05), which was consistent with our predictions and the results of BAT. ELISA inhibition assays indicated that maximal inhibition by P1-P5 was about 20% to recombinant Can f 6, and there is no inhibition by the unrelevant control peptide P6 to recombinant Can f 6, which demonstrated the allergenicity of the B cell epitopes we predicted as well.

In our research, all of the five B cell epitopes are consisted of consecutive amino acids, which means they belong to the sequential B cell epitopes. Our results suggested that sequential epitopes may play an important role in Can f 6-associated dog allergy. Besides, conformational epitopes on allergens are considered to play an important role in initiating human IgE-mediated allergic reactions. So, we will aim to look for the conformational epitopes to research their roles compared with sequential epitopes.

In summary, Can f 6 recombinant protein was expressed, purified and detected the allergenicity by Western blot and basophil activation test. And the Can f 6 protein was homology modelled and its 5 B cell epitopes were predicted and identified by ELISA. This work could provide an efficient strategy for epitope prediction and can be further application for diagnosis and therapeutics.

## MATERIALS AND METHODS

### Patients and Samples

Totally 32 pediatric patients ‘ sera with allergic symptoms with positive SPT results (allergens were supplied by ALK-Abelló, Inc., Hørsholm, Denmark) and with positive serum IgE test results to the dog extract [by using ImmunoCAP assay (Pharmacia Diagnostics AB, Uppsala, Sweden)] were collected. And 6 healthy children sera were collected. A total of 32 pediatric patients, mean age (7.25) years, were enrolled as serum-positive for dog allergen. The patients’ major information were collected and presented in Table 1. In Table 1, the value of ImmunoCAP was obtained. Additionally, the sera of 6 healthy children, mean age (5.75) years, were treated as negative controls. The study protocol was approved by the ethical committee of the First Affiliated Hospital of Nanjing Medical University. Written informed consent for the use of blood samples were obtained from all participants before study entry according to the declaration of Helsinki.

### Sequence retrieval

Can f 6 has 528 bases pairs, encoding 175 amino acids. Can f 6 gene was optimized on the basis of unchanged amino acid sequences [18], according to the GenScript rare codon analysis tool (http://www.genscript.com/.cgi-bin/tools/rare_codon_analysis) and synthesized (GenScript, Nanjing), then subcloned into pET-28a vector with the sites of *EcoR I* and *Xho I* and verified by DNA sequencing.

### Expression and purification of Can f 6 in E. coli

The pET28a-Can f 6 plasmid was transformed into BL21 (DE3) pLysS *E. coli*. A positive colony was selected and incubated at 37°C overnight. All of the broth was inoculated into 300 ml LB- kanamycin broth and incubated at 37°C with shaking at 200 rpm until the optical density (OD) value at 600 nm reached 0.6∼0.8. Isopropyl-b-D -thiogalactopyranoside (IPTG) (Takala, Dalian, China) was added to the broth with the final concentration of 1.0 mM and incubated for another 6 h. The bacterial cells were gathered by centrifugation at the speed of 3,600 × g at 4°C for 30 min, and the sediment was harvested. The sediment was lysed in a lysis buffer by sonication at 20 kHz, 5 sec pulse-on, 5 sec pulse-off. After collected by centrifugation at 12,000 × g at 4°C for 30 min, the supernatant was loaded on the nickel column (Genscript, Nanjing, China), washed with running buffer containing 500 mM NaCl, 50 mM NaH_2_PO_4_, 10 mM imidazole, pH 8.0, and eluted in order with elution buffer containing 500 mM NaCl, 50 mM NaH_2_PO_4_, 30, 50, 100, 150, 200, 250 mM imidazole, pH 8.0, and all of the eluted fractions were collected.

### Immunoreactivity of human sera with recombinant Can f 6

The IgE-binding ability of Can f 6 was detected by Western blot analysis. Recombinant Can f 6 (5 µg) was separated by SDS-PAGE and then transferred to the polyvinylene difluoride (PVDF) membranes (Merck KGaA, Darmstadt, Germany). The PVDF membranes were blocked in 5 % skim milk for 3 h, then incubated with thirty-two sera of dog allergic children [diluted 1:20 in Tris-buffered saline tween (TBST)] as the first antibody overnight at 4°C. Following washed with TBST, the membranes were incubated with horse radish peroxidase- conjugated goat anti-human IgE monoclonal antibody (KPL, Caithersburg, USA). The positive protein bands were visualized by incubating the membranes with tetramethylbenzidine peroxidase substrate. Four healthy children serum were used as negative control.

### Basophil activation test (BAT)

Allergens can activate basophils by inducing the expression of CD63 and CCR3 on the basophil surface, which is considered as the indicator of basophil activation [19]. In brief, peripheral blood mononucleated cells (PBMCs) from 20 ml blood collected by healthy persons were separated by Ficoll-Paque density gradient, and treated with 10 ml LS (a solution containing 1.3 M NaCl, 0.005 M KCl and 0.01 lactic acid, pH 3.9) for 2 min at 8°C. Following neutralization with 12 % Tris (pH 10.9), non-specific IgE on the basophils was wiped off and the cells were sensitized with the sera of 6 children with Can f 6 allergies confirmed by WB and they have the highest ImmunoCAP value, or the 6 healthy controls (1 in 10 dilution, 2 h at 37°C) (same patients and controls as mentioned above) as previously described. Then, the cells were challenged with the Can f 6 (1.0 µg/ml) for 15 min at 37°C. The goat anti-human IgE antibody (Serotec, Kidlington, UK) was used as a positive control. The anti-human CD63-FITC antibody (HH-MHCD63014; Invitrogen) and CCR3-PE-labeled antibody (85-12-1939-42; eBioscience Inc., San Diego, CA, USA) were added for 15 min at 37°C. Flow cytometric analysis of surface markers was performed at 488 nm on a FACSAria flow cytometer (Becton-Dickinson, Franklin Lakes, NJ, USA) and analyzed by FACSDiva software.

### Homology modeling

Can f 6 modeled protein structure was built through alignment mode in SWISS-MODEL. The homologous template, which was suitable for the Can f 6, was selected from SWISS-MODEL server [20, 21].

### Secondary structure prediction

The transmembrane helices of Can f 6 was predicted by TMHMM 2.0 [22]. The secondary structure (α-helices, β-sheets and random coils) for Can f 6 were predicted by PyMOL molecular graphics system [23].

### Prediction of B cell epitopes

DNAStar protean system, BepiPred 1.0 server and BPAP system were used to predict the B-cell epitopes of Can f 6 [24, 25]. The ultimate results were obtained by the combined results of the three tools together [26, 27].

### Peptide synthesis

The five predicted B-cell epitopes and an unrelevant control peptide of Can f 6 (P6 = VVHDGYNLFR) were synthesized by GenScript (Nanjing, China) with the purity of ≥ 90% and the sequences were confirmed by mass spectrometry (MALDITOF) analysis.

### Verification of B cell epitopes by Enzyme linked immunosorbent assay (ELISA)

ELISA was used to detect the specific IgE binding of the predicted B cell peptides [28]. The 96-well microplate (Costar, USA) were coated with 2 μg/100 μl/well (0.02μg/μl) of predicted peptides in Phosphate Buffer Saline (PBS, pH 7.4) overnight 4°C. The plates were washed with PBS-T (PBS containing 0.1% Tween 20) (200μl/well) three times. The plates were then blocked with Bovine Serum Albumin (BSA, 1 mg/ml) (200 μl/well) for 1 h at 25°C and washed with PBS-T once. And then incubated with 100 μl of 6 Can f 6 allergic children’s sera (same as the BAT) (1:100 v/v in PBS) at 25°C for 2 h. Post incubation the plates were washed with PBS-T three times. The plates were incubated with 1:4000 v/v diluted horse radish peroxidase- conjugated goat anti-human IgE monoclonal antibody (KPL, Caithersburg, USA) at 25°C for 1 h and washed with PBS-T three times, followed by 100 μl of a TMB Color liquid (KeyGEN BioTECH, Nanjing, China) were added to each well. Thirty minutes later after coloration, the reaction was stopped by adding 50 μl of 3 M H_2_SO_4_, Sera from 6 healthy subjects without a history of allergic symptoms were used as control normal human serum (NHS). After incubation the plates were processed as described and absorbance was read at 450 nm (Biotech Eon, Biotech, USA).

### Inhibition ELISA experiments of 5 B-cell epitopes

Inhibition ELISA experiments of Can f 6 were detected with 5 B-cell peptides, P6 and recombinant Can f 6 in two dog allergic children. The 96-well microplate (Costar, USA) were coated with 2 μg/100 μl/well (0.02 μg/μl) of predicted peptides in PBS overnight 4°C. The plates were washed with PBS-T (PBS containing 0.1% Tween 20) (200 μl/well) three times. The plates were then blocked with bovine werum albumin (BSA, 1 mg/ml) (200 μl/well) for 1 h at 25°C and washed with PBS-T once. And then incubated with 100 μl of 2 allergic children’s sera (1:100 v/v in PBS) which have been precoated with P1-P6 and recombinant Can f 6 for 1 h at 37°C with the 6 gradient concentrations (0.001,0.01,0.1,1,10,100μg/ml) at 25°C for 2 h. Post incubation the plates were washed with PBS-T three times. The plates were incubated with 1:4000 v/v diluted horse radish peroxidase- conjugated goat anti-human IgE monoclonal antibody (KPL, Caithersburg, USA) at 25°C for 1 h and washed with PBS-T three times, followed by 100 μl of a TMB color liquid (KeyGEN BioTECH, Nanjing, China) were added to each well. Thirty minutes later after coloration, the reaction was stopped by adding 50 μl of 3 M H2SO4, Sera from 6 healthy subjects without a history of allergic symptoms were used as control normal human serum (NHS). After incubation the plates were processed as described and absorbance was read at 450 nm (Biotech Eon, Biotech, USA).
